# Kinesin motor density and dynamics in gliding microtubule motility

**DOI:** 10.1038/s41598-019-43749-8

**Published:** 2019-05-10

**Authors:** Virginia VanDelinder, Zachary I. Imam, George Bachand

**Affiliations:** Center for Integrated Nanotechnologies, Sandia National Laboratories, Albuquerque, 87185 NM USA

**Keywords:** Nanoscale biophysics, Nanoscience and technology

## Abstract

Kinesin motors and their associated filaments, microtubules, are essential to many biological processes. The motor and filament system can be reconstituted *in vitro* with the surface-adhered motors transporting the filaments along the surface. In this format, the system has been used to study active self-assembly and to power microdevices or perform analyte detection. However, fundamental properties of the system, such as the spacing of the kinesin motors bound to the microtubule and the dynamics of binding, remain poorly understood. We show that Fluorescence Interference Contrast (FLIC) microscopy can illuminate the exact height of the microtubule, which for a sufficiently low surface density of kinesin, reveals the locations of the bound motors. We examine the spacing of the kinesin motors on the microtubules at various kinesin surface densities and compare the results with theory. FLIC reveals that the system is highly dynamic, with kinesin binding and unbinding along the length of the microtubule as it is transported along the surface.

## Introduction

Cytoskeletal motor proteins and their associated filaments play a vital role in many cellular processes, including mitosis and meiosis, actuation of cilia, and trafficking of cargo within cells^1^. For example, cargoes (e.g., organelles) bound to the motor protein kinesin-1 (KFI3B; henceforth referred to as simply ‘kinesin’) are transported throughout the cell as the motors walk along a network of microtubules filaments^1^. For close to two decades, this system has been broadly used in nanotechnology applications in which the components are reconstituted *in vitro*^2,3^. In particular, they have served as one of the primary systems for studying active self-assembly phenomena^4^, as well as developing bioanalytical assays based on motor-protein transport^5^. For such *in vitro* applications, the geometry of the assay is most commonly inverted, with surface-attached motor proteins transporting microtubules around (commonly referred to as an inverted or gliding motility assay).

Despite its long history and widespread use, basic parameters of the gliding motility assay, for example how many kinesins are bound to and transport a microtubule, remain unclear. To address the issue of how many kinesin are bound, Kabir *et al*. used two different techniques with kinesin attached to PDMS and then compressing the PDMS to bend the microtubules into a sinusoidal shape, with the period correlating to the spacing of the bound kinesin^6^; or pulling on the PDMS so the microtubules broke into pieces with the length of the pieces correlating to the spacing of the bound kinesin^7^. These experiments demonstrated that the spacing of bound kinesin was inversely proportional to the kinesin surface density. However, these experiments only accessed the spacing of bound kinesins on stationary microtubules, which may not correlate directly to the number bound during active transport.

When microtubules are moved across a surface by kinesin, there is the potential for the number of kinesins bound not to follow an inverse relationship with the kinesin surface density due to effects from the moving microtubules. Duke, Holy, and Leibler developed a theory (DHL) of how the average spacing of motors on filaments, <d>, should vary with respect to motor surface density, *σ*^8^. At high kinesin surface density (regime I, Fig. S1), the free tip of the microtubule filament does not have time to explore the surface before encountering a motor, so the area explored by the filament tip is governed by the capture radius of motor, *w*, which has been estimated to be between 80–90 nm^9^. Here, the average spacing of the motors will be <d> ~ *σ*^−1^*w*^−1^. At the medium surface density (regime II), thermal fluctuations lead the filament tip to sweep the surface; the area swept by the filament depends on the filament persistence length, p. In regime II, it is predicted that <d> ~ *σ*^−2/5^*p*^1/5^. At the low surface density (regime III), the forward motion of the microtubule (average speed ν) prevents the tip from having enough time to search all of the thermal fluctuation space, so regime III is instead dominated by the rotational diffusion coefficient, *γ*. Then in regime III, <d> ~ *σ*^−1^(*γ*/ν)^−1/^^2^. DHL theory contends that this regime does not exist in practice due to the filament leaving the surface before finding another motor. Using rough estimates for these physical parameters for kinesin and microtubules, DHL theory predicts that the transitions from regime I to II and regime II to III occur around ~20 kinesin μm^−2^ and 0.05 kinesin μm^−2^, respectively. DHL theory assumes that the filaments are confined to 2D, which is not the case in experimental inverted motility assays. A further limitation of DHL theory is that it assumes that binding of new motors only occurs at the filament tip and does not consider motors binding or coming unbound along the length of the filament. As the typical run length of an individual kinesin along a microtubule is 1070 ± 30 nm, which is much shorter than length of microtubules, many binding and unbinding events are expected^10^.

To study the relation between the average spacing of motors along filaments and the motor surface density, it is necessary to be able to measure both values. In 1989, Hancock and Howard estimated the kinesin surface density by assuming that all of the kinesin in the solution in the glass flow cell bind to the top and bottom surfaces^11^. Thus, by knowing the dimensions of the flow cell and the initial kinesin concentration, the approximate kinesin surface density may be estimated. Estimates of kinesin surface density using the Howard method, however, were shown to differ by an order of magnitude than the density predicted by the DHL theory^8^. In order to accurately measure the kinesin surface density, a method based on microtubule landing rates was developed^12,13^. Attaching fluorescence markers (dyes or quantum dots) to kinesin combined with single molecule imaging techniques would offer the possibility to directly visualize the kinesin surface density. However, this requires modified kinesin constructs and different surface attachment methods from typical adhesion-based motility assays. GFP-kinesin conjugates are problematic to image for tracking at the single molecule level due to poor photostability^14^. Tjioe *et al*. recently developed a method to rapidly conjugate quantum dots with kinesin and purify them using magnetic beads, and they demonstrate visualization of the locations of kinesin attached to the surface^15^.

A direct measurement of the kinesin motor spacing on motile microtubules was performed by Fallesen *et al*., in which optical tweezers were used to pull at the lagging end of a microtubule at a 90° angle to the direction of motion: a jump in the location of the vertex of the angle indicated the location (disassociation) of a motor^16^. Fallesen *et al*. also observed an order of magnitude disparity between the kinesin surface density predicted by Howard’s absorption method and the results of their optical tweezer measurement of <d>. They reported that the average motor spacing followed approximately −1/2 power, similar to the −2/5 predicted by DHL theory. However, they could only image one spacing length at a time, not all the kinesin bound to the microtubule simultaneously, thus missing any potential dynamics of kinesin binding/unbinding. Van den Heuvel *et al*. measured the distance between pivot points in a microtubule’s trajectory, along with the DHL theory^8^, to calculate the length of the leading tip, which can be considered equivalent to <d> if no subsequent binding or unbinding of kinesin occurs along the length of the microtubule^17^. Van den Heuvel *et al*. used the measurement of the <d> in conjunction with trajectories measured under perpendicular electrical forces at a single kinesin surface density to calculate the tip persistence length of the microtubule tip to be 0.08 ± 0.02 mm, which is much smaller than the 4–8 mm values measured for long microtubules. Tjioe *et al*. were able to detect when the kinesin is attached to a microtubule by deviation of the kinesin from its rest position that was possible to resolve due to the long 1565 bp DNA linker between the surface and the kinesin^15^. However, they did not investigate the spacing of the kinesin on the microtubules or the effect of kinesin surface density on spacing. Despite all of these findings, fundamental questions regarding the gliding motility (e.g., number and dynamics of kinesins participate in microtubule transport) remain unanswered.

In the present paper, we report the use of fluorescence interference contrast microscopy (FLIC) to image the locations where kinesins are bound to a microtubule while the filament is transported. Previously, this method has been shown to be effective for determining the location of myosin motors on actin filaments^18^. Using the FLIC method, we studied the effect of kinesin surface density on the average spacing of bound kinesin, and compared those results to kinesin surface density measured using microtubule landing rates. Here, we observed kinesin binding and unbinding along the entire length of microtubules during gliding, revealing that the process is more dynamic than previously considered.

## Results and Discussion

### FLIC observation of inverted motility assay

**Figure 1:**
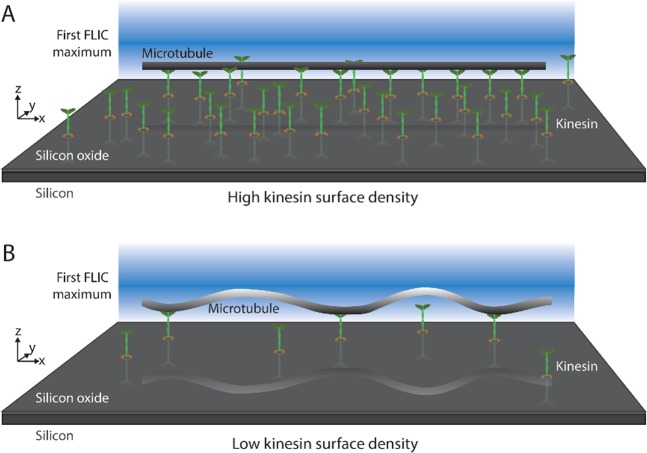
Cartoon (not to scale) of a microtubule in gliding motility assay with high (**A**) and low (**B**) kinesin surface density. In the low-density example, the grey level of the microtubule corresponds to the relative fluorescence signal in FLIC microscopy.

**Figure 2:**
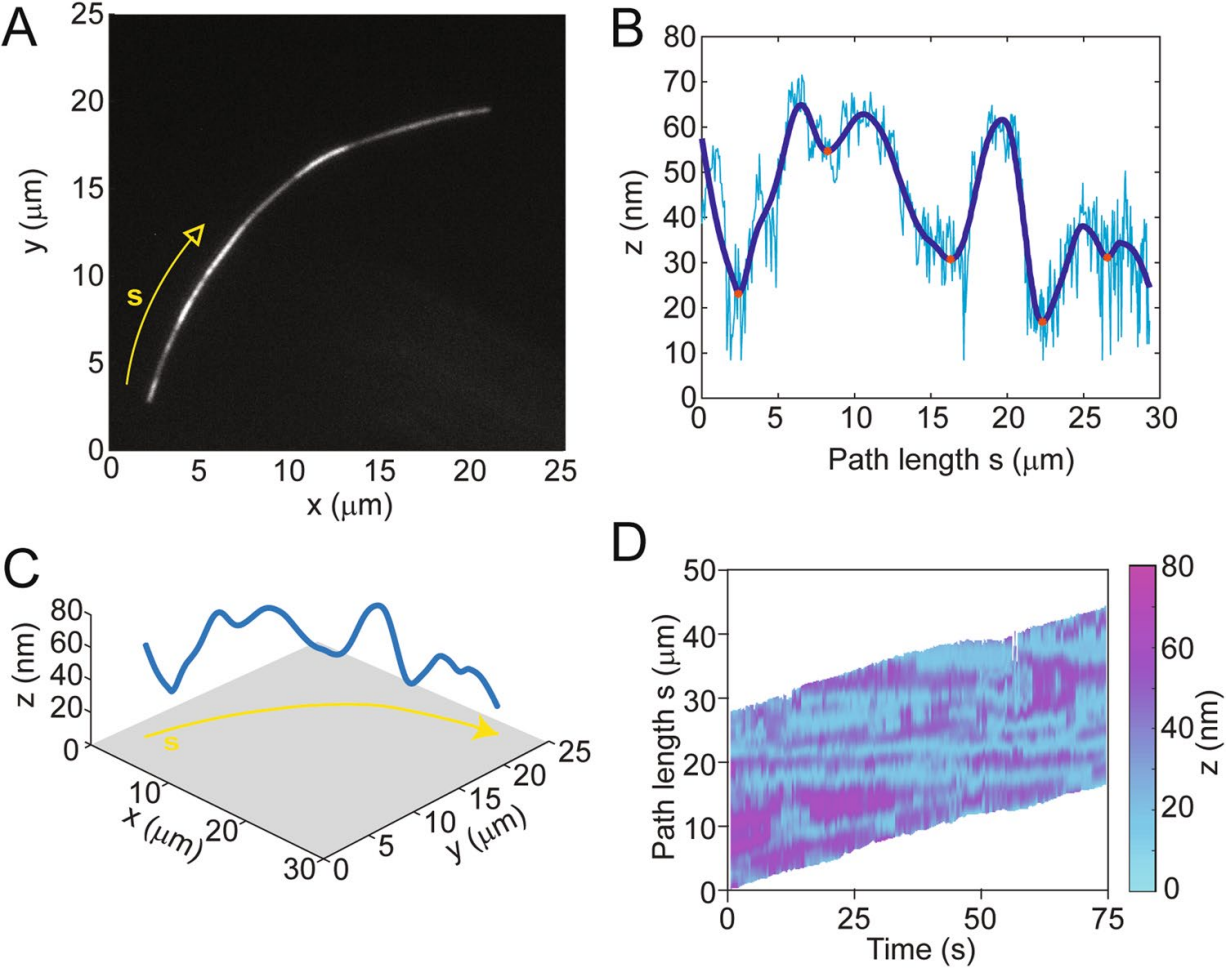
(**A**) Fluorescence micrograph of microtubule gliding along silicon surface with FLIC setup. Arrow indicated direction of motion of the microtubule along the path *s*. (**B**) Height *z* of the microtubule away from surface along path s. (**C**) 3D plot of microtubule in *x* (μm), *y* (μm), and *z* (nm). (**D**) Kymograph of height *z* of microtubule along path s for microtubule over the course of time.

FLIC reveals the distance of a fluorescent object away from a silicon surface as variations in intensity in a sinusoidal manner with a periodicity of ~230 nm (for TRITC). The FLIC intensity was converted to distance *z* using the method of Kerssemaker *et al*.^19^; details are provided below in the methods section. The dimensions of FLIC sensitivity make it an excellent tool for studying kinesin/microtubule motility assays, as the stalk on the kinesin are ~80 nm in length and the microtubule is ~22 nm in diameter. Using FLIC, the kinesin construct used in the present work, KIF3B, was shown to hold microtubules a distance of 16.8 ± 1.9 nm away from the surface^19^. This distance is much shorter than the length of the kinesin stalk, but has been explained by the kinesin stalk functioning as a spring^20^. At high kinesin surface density, the entire microtubule is equidistant from the surface and appears uniform in intensity (in this case, the microtubule is near the FLIC minimum and appears dim, Fig. 1A). Previous observations of kinesin/microtubule motility assays with the FLIC technique were performed under this condition. However, at a sufficiently low kinesin surface density, FLIC demonstrates that parts of the microtubule are further from the surface than others (indicated by an increase in brightness in FLIC, Figs 1B, 2A). The intensity of fluorescence from the microtubules is between the measured minimum and maximum FLIC intensities, which indicates that the microtubule lies within the first FLIC half period and that there is a one-to-one correspondence between intensity and distance *z* from the surface. (Near the ends of the microtubules the intensity sometimes reaches the FLIC maximum, which indicated that the tip of the microtubule is further than half a FLIC period (115 nm) away from the surface and the 1:1 correspondence between intensity and *z* is broken at the microtubule tip.) The buckles in the microtubules were regions between bound kinesin that extend up to ~70 nm from the surface. For a representative microtubule, the distance *z* that the microtubule is away from the surface along its path is shown in Fig. 2B; a 3D plot of the same microtubule is depicted in Fig. 2C.

**Figure 3:**
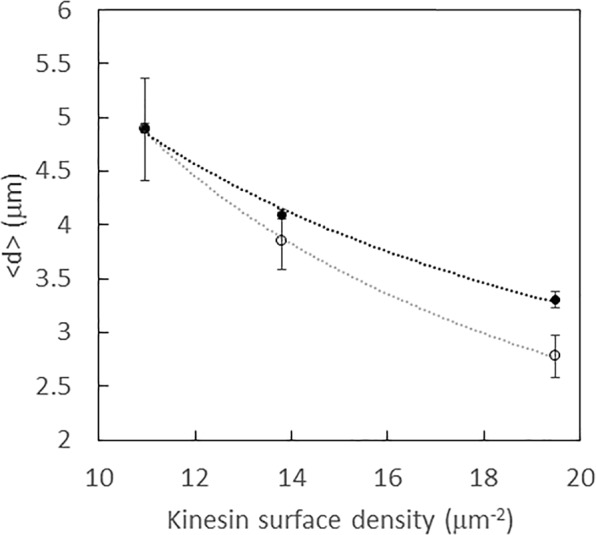
Relation between <d> and *σ* (as measured with microtubule landing rate experiments) for both mobile (solid circles) and immobile (rings) microtubules. Dotted lines show a power law fit to the data. Error bars denote standard error of the mean.

For immobilized microtubules that settled on the surface of the flow cell in the presence of AMP-PNP, a non-hydrolysable analog of ATP, the spacing of the intensity minima is inversely proportional to the surface density of the kinesin, as shown in Fig. 3. The intensity minima, which indicate points where the microtubule is close to the silicon surface, reveal locations where the kinesins are bound to the microtubule. Examining moving microtubules exhibits further evidence that the FLIC minima reveal the location of bound kinesin. Specifically, when observing gliding microtubules, the location of the minimum fluorescence intensity points remain in the same place as the microtubule is transported along, as shown in Fig. 2D. It is possible that a fraction of the minima that persist for only a single frame may correspond to thermal fluctuations of the microtubule closer to the surface rather than indicating the location of a kinesin motor. While fluctuations may slightly overestimate the number of bound kinesin, the fluctuations should be independent of the kinesin surface density and thus not affect comparisons between different kinesin surface densities.

Using FLIC, Krenc *et al*. were able to observe the locations of myosin motors on gliding actin filaments^18^. They showed that myosin holds actin 10 nm above the surface and studied the size of buckles (where the actin filament was up to 60 nm away from the surface) to determine coordination among motors. Their work supports our conclusions on the viability of the FLIC technique to locate bound motor proteins on filaments in gliding assays.

Unlike the optical tweezer technique used by Fallesen *et al*.^16^, which only shows location of the kinesin bound closest to the tail of the microtubule, the FLIC method shows all points where kinesin are bound to the microtubule simultaneously. The FLIC method, however, only works within a limited range of surface kinesin density: if too many kinesin are bound, then the locations of the FLIC minima get too close and there are no observable changes in height. At very low kinesin surface density, the spacing between kinesin becomes larger than the microtubule length, preventing transport (regime III of DHL theory).

The method of Tjioe *et al*. to purify quantum dot conjugated kinesin could potentially be able to access some of the same data as the FLIC assay in order address questions of cooperativity of kinesin in a motility assay. In addition to the bound points between kinesin and microtubules, they can determine if the kinesin is hindering or helping the motion of the microtubule^15^. However, the method of Tjioe *et al*. requires a very long linker, which would impede studies of motor cooperativity. The FLIC system, on the other hand, requires no modifications of the kinesin or linker, making it a closer match to the systems presented in literature.

### Effect of kinesin surface density on kinesin spacing

The amount of kinesin introduced to the glass/silicon flow cell was varied linearly by changing the concentration of the kinesin solution, as listed in the first column of Table 1. From the dimensions of the flow cell and the kinesin concentrations, the method of Howard and Hancock^13^ was used to estimate the surface density of the kinesin, *σ*, which are listed in the second column of Table 1. The surface density was also measured using microtubule landing rates^12^ (Table 1, third column), as shown in Fig. S2 and described in the methods section. The relation between the initial kinesin concentration in solution and that measured adsorbed to the surface are consistent with previous estimates^21^.

**Table 1:** The concentration of the initial kinesin solution, the nominal and measured kinesin surface density, distance between bound points for motile and immobilized kinesin, and the calculated kinesin surface density at five different kinesin concentrations.

[kinesin] (nM)	Nominal *σ* (kinesin/μm^2^)^a^	Measured *σ* (kinesin/μm^2^)^b^	<d> (μm) motile^c^	<d> (μm) immobilized^c^	Calculated *σ* (kinesin/μm^2^)^d^
3.6	44	19	3.3 ± 0.08	2.8 ± 0.2	7.3
2.9	35		3.9 ± 0.03	3.3 ± 0.2	4.4
2.4	29	14	4.1 ± 0.04	3.9 ± 0.3	4.1
2.1	25		4.2 ± 0.05	4.7 ± 0.3	4.0
1.8	22	11	4.9 ± 0.1	4.9 ± 0.5	3.3

±values indicate standard error of the mean. The number of measurements of <d> for motile microtubules were 554, 3550, 2996, 1649, and 2719 for the [kinesin] = 3.6, 2.9, 2.4, 2.1, and 1.8 nM, respectively. The number of measurements of <d> for immobilized microtubules were 47, 36, 36, 46, and 25 for the [kinesin] = 3.6, 2.9, 2.4, 2.1, and 1.8 nM, respectively.

^a^Estimated based on method of Hancock and Howard^13^.

^b^Determined by microtubule landing rate experiments^12^.

^c^Measured with FLIC.

^d^Calculated from <d> following Fallesen metho^d16^ based on DHL theory^8^.

The dependence of the average spacing of bound kinesin, <*d*>, on microtubules was examined for both mobile and immobilized microtubules (Fig. 3, Table 1) as a function of *σ*, which was determined via landing rate measurements (Fig. S2). Although the FLIC technique is limited to a small range for *σ*, we performed a power law fit to the data consistent with similar relationships established in prior theory studies. For the immobilized microtubules, we found that <*d*> ~ *σ*^−1^ (R^2^ of fit = 0.98), which is the expected exponent reported by Kabir *et al*.^6^. For motile microtubules, in the range of kinesin surface density used in these experiments, <*d*> ~ *σ*^−0.6^ (R^2^ of fit 0.91), which higher than the exponent value of −0.4 predicted by the DHL theory for regime II. The only other measurement of <d> on mobile microtubules, as measured with optical tweezers by Fallesen, found the exponent to be −0.5. Given the small range of *σ* measured, the data presented by Fallesen *et al*. is in reasonable agreement with the DHL theory for regime II, while our data lies in between what is expected for regime I and II.

Further, DHL can be expanded to predict a probability distribution *R*(*x*) for *d*, with the sole variable in the equation being the kinesin surface density: $R(x)=\exp (\,-\,6.2\,\sigma \,{x}^{5/2})\{15.4\,\sigma \,{x}^{3/2}\}$, the derivation of which is given in Fallesen *et al*.^16^. This distribution can be fitted to a histogram of *d* values (Fig. S3) and used as another way to calculate *σ*. Values obtained from this method are listed in the last column of Table 1. The three different methods of determining *σ* resulted in different values: the nominal *σ* calculated using the Howard method was a factor of 2 higher than that measured with landing rate and a factor of 7 higher than calculated from the distribution of *d*. The Howard method might predict a higher *σ* value than the landing rates because not all the kinesin might adhere to the surface in a configuration that can bind to microtubule. The lower value of *σ* calculated from the distribution of d suggests that the microtubule does not bind to all the kinesin that are present along its trajectory during gliding.

### Effect of microtubule persistence length on kinesin spacing

According to DHL theory, in Regime II, <d> should have a dependence on the persistence length *p* to the 1/5 power^8^. The persistence length of the microtubules may be adjusted in a controlled manner between ~100 and 350 μm by adding BaCl_2_ to the solution^22^. We used FLIC to measure <d> of kinesin bound to the microtubules at the same surface density *σ*, but across a range of persistence lengths. As shown in Fig. S4, no dependence of <d> on *p* was observed. As a 1/5 power dependence is rather small, it is possible that the data was too noisy or the range of persistence lengths tested was too small to observe the dependence. A lack of dependence on *p* could also arise from the *σ* being too high, such that the system is on the edge between regimes I and II. Regime I is not theorized to have a dependence of <d> on *p*. Alternatively, van den Heuvel *et al*. showed that the persistence length of the tip of the microtubule is only 0.08 ± 0.02 mm, rather than the established value of ~4–8 mm measured for long microtubules^17^. The experiments of Bouxsein *et al*. measuring the persistence length of the microtubule in the presence of BaCl_2_ looked at long microtubules. As it has been theorized that microtubules behave as a loose assembly of independent protofilaments on short length scales^23^, it is possible the persistence length of the microtubule tip does not scale the same as the long microtubule in the presence of BaCl_2_.

### Dynamics of kinesin binding/unbinding

**Figure 4:**
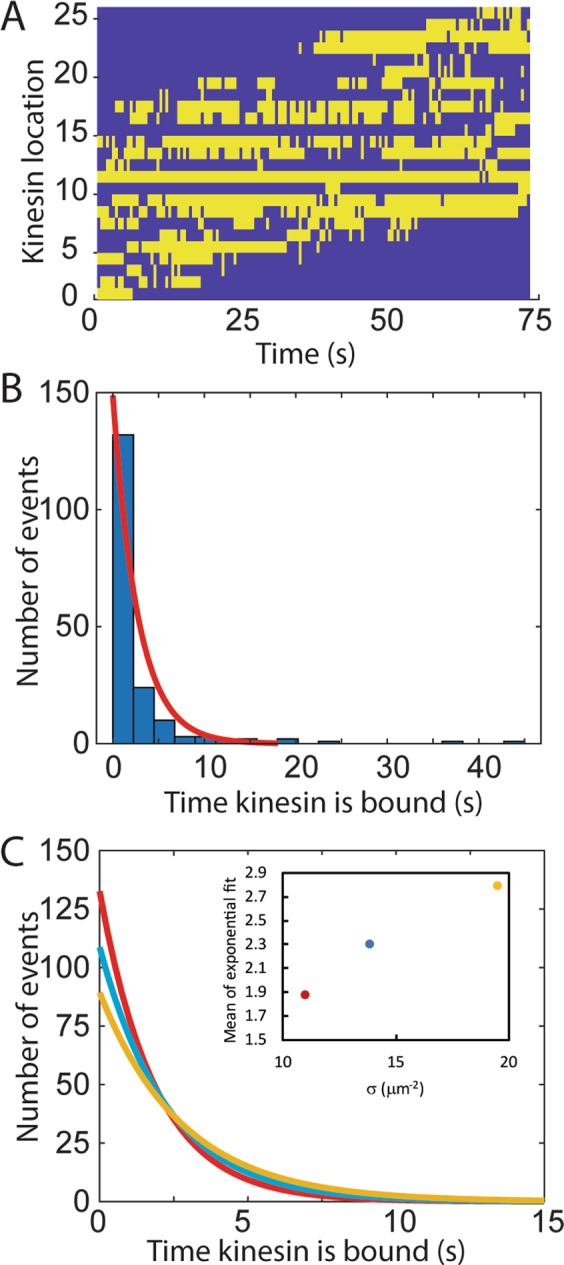
(**A**) The presence (yellow) or absence (blue) or a kinesin at each kinesin location (intensity minimum) is plotted as a function of time. (**B**) The number of seconds that kinesins are bound to the microtubule displays an exponential distribution, with an exponential fit to the curve shown in red. (**C**) The exponential fits for the various kinesin surface density (*σ*) conditions (red = 11 μm^−2^, blue = 14 μm^−2^, yellow = 19 μm^−2^). Inset shows the mean of the exponent versus (*σ*).

The power of the FLIC technique is its ability to observe where all kinesin motors are bound to the microtubule simultaneously. Some kinesin motors are observed to stay bound for the whole length of the microtubule (>75 s), whereas others are observed to transiently bind and unbind (Fig. 4A). This transient binding and unbinding is not addressed in the original DHL theory, which assumes that binding only occurs at the leading tip. As shown in Fig. 4B, the number of frames that a kinesin is bound to the microtubule follows an exponential distribution (adjusted R^2^ = 0.99). The average attachment time of kinesin on the microtubule (shown in Fig. 2A) was measured to be 2.1 s, which corresponds to approximately 60 steps. (For comparison, the average run length of an individual kinesin motor walking on a microtubule was measured by Verbrugge *et al*. to be 1070 ± 30 nm, or 133 steps)^10^. The average attachment time as a function of *σ* is shown in the inset in Fig. 4C. The distribution of attachment times might be due to the positioning of the microtubule relative to the kinesin on the surface. The linker on the kinesin has been proposed to function as a spring. Kinesin that are positioned directly beneath a microtubule may have a larger attachment time because they do not have strain in the linker, whereas kinesin that are slightly off-center and must stretch the linker to reach the microtubule might have shorter attachment times with more binding and unbinding events. This effect has been described in simulations in which the binding probability of surface-adhered kinesins was evaluated based on the distance between the microtubule axis and the kinesin attachment point^9^. Variations in linker positions might also explain the variations in the height of the bound minima as shown in Fig. 2B.

The attachment times are in agreement with Gibbons *et al*., who developed a stochastic model of kinesins and microtubules treating kinesin motors as springs^20^. In their model, examination of attachment times demonstrated that individual motors could detach and reattach multiple times during transport. When considering the cumulative attachment time of each motor, most motors were found to be attached only briefly. In agreement with the data here, changing the surface density of motors in the simulations changed the distribution, with a higher motor density displaying a longer tail of motors with long attachment times^20^.

The number of kinesin bound to the microtubule was also evaluated in relation to the instantaneous velocity of the microtubule transport. No correlation between these two parameters was observed (*r* = −0.0156, P = 0.851); variance in the velocities due to the tip moving out of plane at low motor densities, however, may have limited the accuracy of these measurements. This result was anticipated as the microtubule velocity was not predicted to depend on the number of motors bound in the range of *σ* used in these experiments^20^.

## Conclusions

We used FLIC to observe where kinesin motors were bound to microtubules during gliding motility. The motors were observed to bind and unbind the microtubule in a highly dynamic fashion during transport, which has not been accounted for in prior theory or models of motility. As such, these data provide critical insights that may be used to further refine our understanding of microtubule transport. Our results also confirmed previous observations^8,16^ that the adsorption-based method^11^ of estimating the *σ* is off an order of magnitude. Because the FLIC enables visualization of motor attachment during transport, this method may be further applied to other motor systems (e.g., dynein transport), as well as analyzing the system in the presence of roadblocks and examining the effects on velocity as a function of the number of kinesin bound. The FLIC system would also be useful for studying motor cooperativity. As with myosin and actin, there is a possibility that there is force transduction between kinesin motors through the microtubule. Although the surface-adhered kinesin working cooperatively to transport microtubules is not as common a geometry as myosin/actin, there are still biological parallels, such as actuation of cilia, where a better understanding of kinesin cooperativity would be relevant.

## Methods

### Chemicals, buffers, and supplies

Unless otherwise noted, all chemicals were obtained from Sigma-Aldrich. The silicon flow cell was constructed using a No 1.5 glass coverslip, two pieces of extra thin double-sided tape (10 μm, Nitto Denko), and a 1 by 0.5 cm piece of silicon. All of the silicon pieces used in these experiments was from the same 4″ wafer. The thickness of the native oxide layer on the silicon, *z*_0_, was measured to be 2.89 nm (MSE 0.6148) with a VASE_V ellipsometer.

BRB80 buffer consisted of 80 mM PIPES, 1 mM EGTA, and 1 mM MgCl_2_, pH 6.9. The BRB80CAT buffer consisted of BRB80 buffer with 1 mM ATP, 10 μM taxol, 0.2 mg mL^−1^ casein, and 600 μM DTT (BioRad). For imaging, glucose oxidase and catalase buffer (GODCAT) consisted of BRB80CAT supplemented with 20 μg/mL glucose oxidase, 8 μg/mL catalase, 20 mM D-glucose, and 1 mM Trolox (TX), 100 μM of which was in the oxidant Trolox quinone (TQ) form, as determined from the absorbance spectrum. For experiments with immobilized microtubules, the same solutions were used except substituting adenosine 5′-(β,γ-imido)- triphosphate (AMP-PNP, Sigma), a nonhydrolyzable ATP analog for ATP in the BRB80CAT solution.

### Microtubule and kinesin preparation

Microtubules were made by combining TRITC labelled to unlabeled tubulin (5 mg/mL, Cytoskeleton) in a 1:4 ratio in GPEM buffer, heating at 37 C for 25 minutes, and then stabilizing with BRB80 with 10 μM paclitaxel (Taxol®). KIF3B was purified from *Drosophila* kinesin following previously published protocols^24^. The kinesin concentration was measured with a UV-Vis spectrophotometer (Beckman-Coulter DU640) using ε_280_ = 42500 M cm^−1^.

### Fluorescence interference contrast (FLIC) imaging

The protocol for FLIC experiments was as follows: First, the flow cell was filled with a solution of kinesin in BRB80CAT and allowed to incubate for 5 minutes. Next, the flow cell was washed with a solution of MTs diluted 1:40 in BRB80CAT and allowed to incubate for 5 minutes. Finally, the flow cell was washed with imaging solution and sealed with VALAP. The flow cells were imaged on an Olympus IX-81 microscope with a 100x 1.4NA objective and a Flash 4.0 SCMOS camera.

The protocol outlined in Kerssemaker *et al*. was used to convert between the intensity and the distance z away from the silicon surface^19^. Microtubules in imaging buffer and 0.2% agarose at 50 °C were flowed into a silicon flow cell and allowed to cool to immobilize the microtubules. No kinesin was used; instead microtubules were randomly oriented throughout the flow cell. Microtubules oriented at angles adjacent to the silicon surface were imaged on an Olympus IX-81 confocal microscope, both in confocal and epifluorescence mode. The relation between intensity *I* and height *h* above surface can be approximated by $I(h)={I}_{0}(1-R)\sin \,{}^{4}\pi (\frac{h+{h}_{0}}{{\rm{\Lambda }}})\exp (-\frac{h+{h}_{0}-\frac{1}{2}{\rm{\Lambda }}}{\gamma })+{I}_{0}R$, where *I*_0_ is the maximum fluorescence intensity, *I*_0_*R* is the minimum fluorescence intensity, ${\rm{\Lambda }}$ is the periodicity, *γ* is the decay parameter, and *h*_0_ is a correction for the distance the light travels in the SiO_2_ surface layer. ${h}_{0}=\frac{{n}_{Si{O}_{2}}}{{n}_{{H}_{2}O}}{z}_{0}$, where ${n}_{Si{O}_{2}}$and ${n}_{{H}_{2}O}$ are the indices of refraction of the silicon oxide and water, respectively. However, because the portion of this curve that is of interest in this work is when the height lies between the surface and the first intensity maximum, we can neglect the exponential term in the equation. The periodicity ${\rm{\Lambda }}=\frac{\lambda }{2{n}_{{H}_{2}O}}\approx 230$ nm for TRITC dye. This value was confirmed via confocal microscopy images of the FLIC periodicity for tilted microtubules. The first intensity maximum and minimum were measured from epifluorescence images and were used to calculate *R* to be 25%.

Images were analyzed using ImageJ and Matlab. The RidgeDetection algorithm^25^ was used to locate the MTs in each frame, and the x and y position and intensity of each point along the MT in each frame was exported. A custom Matlab software was used to smooth the intensity profile using a local regression using weighted linear least squares and a 2^nd^ degree polynomial model smoothing algorithm, which was optimized for detecting the positions of the minima. The values of the unsmoothed data at the locations of the detected minima below a set threshold were then identified as locations where kinesin were bound. The program measured the distance between kinesin and the duration for which the kinesin motors were bound.

### Kinesin landing rate measurement

Kinesin landing rates and surface density calculations were performed using a previously established protocol by Katira *et al*.^6^. Briefly, a flow cell filled with a solution of kinesin diluted in immobilized microtubule BRB80CAT was incubated for 5 min. Next, the flow cell was washed with a 4 µg/mL solution of MTs (sheared three times using a 21 G needle from Becton Dickinson, Franklin Lakes, NJ) in imaging solution with AMP-PNP. The flow cell was sealed with VALAP then immediately imaged. Images were collected every 10 seconds, and the time between MT injection and image collection was measured using a digital stopwatch. The number of landed MTs for each frame were counted using ImageJ RidgeDetection then plotted against the time elapsed after injection of MTs. The kinesin landing rate was determined by fitting the following equation:

$$N(t)={N}_{Max}[1-{e}^{(-\frac{Rt}{{N}_{Max}})}]$$

where *N*(*t*) is the number of landed MTs, *t* is time elapsed after MT injection and R is the kinesin landing rate. Kinesin surface density was calculated using the following equation:

$$\rho =-\,\frac{\mathrm{ln}(1-\frac{R}{Z})\,}{A}$$

where $\rho$ is kinesin surface density, *R* is kinesin landing rate, *Z* is the diffusion limited kinesin landing rate assumed to be equal to the landing rate observed at 10-fold dilution from kinesin stock solution, and *A* is MT area, $A=Lw$ assuming a width *w* of 25 nm and average length *L* measured from microtubule images.

## Supplementary information

Supplementary Information
