# Supplementary material for: Kinesin motor density and dynamics in gliding microtubule motility
Source: Sci Rep. 2019 May 10;9:7206. doi: 10.1038/s41598-019-43749-8 (PMC6510761; doi:10.1038/s41598-019-43749-8)
Supplement: Supplementary file 1 — Supplementary Information [file 41598_2019_43749_MOESM1_ESM.docx]

**Supplementary Information**

**Kinesin motor density and dynamics in gliding microtubule motility**

Virginia VanDelinder, Zachary Imam, George Bachand

**
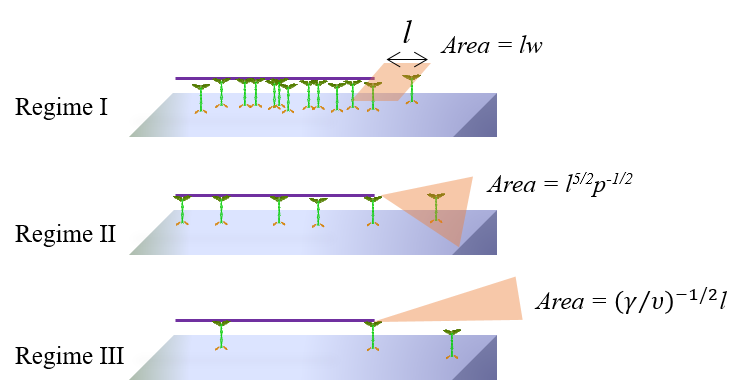
**

**Figure S1.** Cartoon depicting the various regimes of DHL theory. In regime I, the kinesin are so dense that the bound kinesin spacing is governed by the capture radius of the kinesin. In regime II, the microtubule tip can bend and explore the

**
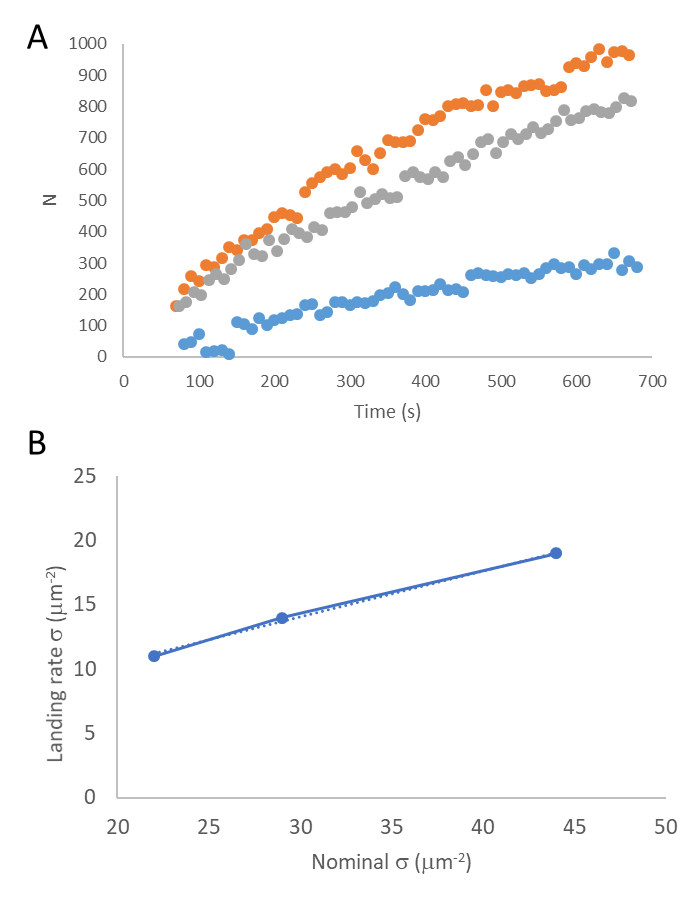
**

**Figure S2.** (A) Number N of microtubules landed on the silicon surface as a function of time. Orange circles, grey circles, and blue circles are at 1:99, 1:199, and 1:799 dilution of the kinesin stock solution, respectively. These curves are fitted to get rate constants R for each concentration. (B) The σ derived from landing rates plotted versus that calculated from the original Howard adsorption method, as described in the methods section.

**
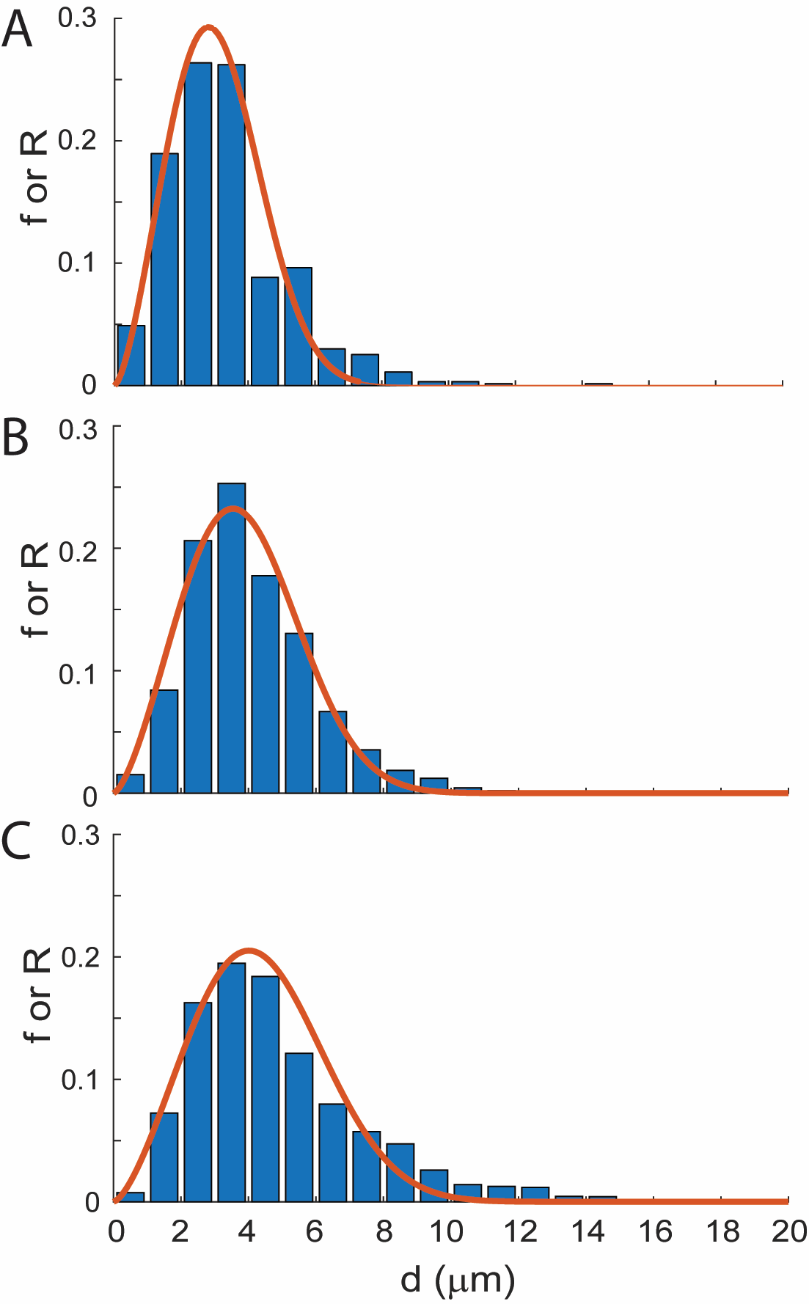
**

**Figure S3.** Measured and calculated distributions of spacing *d* at σ = 19, 14, and 11 kinesin/μm^2^ in A, B, and C, respectively. The experimental data are shown as a normalized histogram frequencies f with bin spacing of 1 μm. The red lines show the normalized probability distribution R calculated by the extended DHL model of Fallesen et al.

**Figure S4.** Average spacing <d> between bound kinesin at various concentrations of BaCl_2_. The persistence lengths of microtubules were measured to be 98, 120, 220, and 357 μm at 0.05, 0.1, 0.5, and 1 mM BaCl_2_, respectively. Error bars denote standard deviation. No dependence of <d> on persistence length was observed.
